# A real-world disproportionality analysis of ripretinib data mining of the public version of FDA adverse event reporting system

**DOI:** 10.3389/fphar.2025.1469597

**Published:** 2025-03-18

**Authors:** Yingkai Feng, Xinyu Fa, Yifei Wang, Tao Zhang, Xuan Sun, Faping Li

**Affiliations:** ^1^ Department of General Surgery, Qingdao Hospital, University of Health and Rehabilitation Sciences (Qingdao Municipal Hospital), Qingdao, China; ^2^ Department of Hematology, Qingdao Rehabilitation Sciences (Qingdao Municipal Hospital), Qingdao, China; ^3^ Department of Gastric and Colorectal Surgery, General Surgery Center, The First Hospital of Jilin University, Changchun, China; ^4^ Department of Urology, The First Hospital of Jilin University, Changchun, China

**Keywords:** FAERS, ripretinib, adverse events, gastrointestinal stromal tumor, pharmacovigilance

## Abstract

**Background:**

Tyrosine kinase inhibitors (TKIs) are the preferred targeted therapy for advanced gastrointestinal stromal tumors (GIST). Ripretinib, the first tyrosine kinase switch control inhibitor, has not yet been extensively studied for long-term safety in large populations. This study evaluates Ripretinib-related adverse events (AEs) in real-world applications by analyzing data from the FDA’s Adverse Event Reporting System (FAERS).

**Methods:**

To quantify signals of AEs, we employed several disproportionality analyses: the Reporting Odds Ratio (ROR), Proportional Reporting Ratio (PRR), Bayesian Confidence Propagation Neural Network (BCPNN), and Multi-item Gamma Poisson Shrinker (MGPS).

**Results:**

In the FAERS database, out of 7,064,646 reports, 3,161 were identified as related to Ripretinib AEs, with 438 significant disproportionality in preferred terms. The most common adverse reactions were tiredness, hair loss, nausea, constipation, diarrhea, loss of appetite, palmar-plantar erythrodysesthesia syndrome, and vomiting. These reactions align with the medication instructions and reports from corresponding clinical trials. Notably, the label includes unexpected and significant AEs such as “hepatic neoplasm”, “hair texture abnormal”, “metastases to liver” and “red blood cell count decreased”. The median onset time for Ripretinib-related AEs was 99 days, with an interquartile range of 27–245 days. Most cases (26.74%, n = 165) occurred within the first month of Ripretinib administration.

**Conclusion:**

Our findings align with clinical observations. We identified novel and unexpected AEs signatures of Ripretinib, indicating that prospective clinical studies are necessary to confirm these findings and clarify their implications. These results could provide valuable evidence to guide further safety studies on Ripretinib.

## 1 Introduction

Gastrointestinal stromal tumors (GIST) are the most common mesenchymal tumors of the gastrointestinal tract, primarily driven by mutations in the KIT (∼80%)and Platelet-Derived Growth Factor Receptor Alpha (PDGFRA) (∼5%–10%) genes (Corless et al., 2011; Heinrich et al., 2003). Tyrosine kinase inhibitors (TKIs) are the preferred targeted therapy for advanced GIST (Blanke et al., 2008; von Mehren et al., 2022). Despite effective first-line treatments like imatinib, and subsequent therapies with sunitinib and regorafenib, resistance often develops due to secondary mutations in the KIT and PDGFRA genes (Liegl et al., 2008), leading to disease progression (Casali et al., 2017; Serrano et al., 2019; Demetri et al., 2006). Ripretinib is a broad-spectrum tyrosine kinase inhibitor targeting KIT and PDGFRα, acting through a unique dual-switch control mechanism (Smith et al., 2019). This mechanism controls the switching of KIT and PDGFRA kinase activation, thereby broadly inhibiting both primary and resistant mutants.

The FDA approved Ripretinib in May 2020 for treating adults with advanced GIST who have previously received three kinase inhibitors (U.S. Food and Drug Administration FDA, 2023). In multiple clinical trials (Blay et al., 2020; Bauer et al., 2022; Li et al., 2024), Ripretinib has demonstrated promising efficacy and favorable safety and tolerability profiles. The most commonly reported AEs for Ripretinib include alopecia, myalgia, nausea, fatigue, and Palmar-plantar erythrodysaesthesia syndrome. In addition, although rare, fatal adverse effects have been reported, such as *de novo* cutaneous squamous cell carcinoma and melanoma (Blay et al., 2020). Therefore, it is crucial to analyze the adverse drug events associated with Ripretinib before its clinical implementation.

The FDA Adverse Event Reporting System (FAERS) is a publicly accessible spontaneous reporting system that collects millions of adverse event reports from healthcare professionals, manufacturers, and other sources (Zhang et al., 2024). Presently, FAERS stands as the largest pharmacovigilance database globally, demonstrating its efficacy in identifying adverse drug reactions (ADRs) linked to drug exposure (Jiang et al., 2024; Zhao et al., 2024; Wang et al., 2024; Yang et al., 2023; Zhong et al., 2024). This study aims to conduct a comprehensive retrospective analysis of ripretinib-related AEs reported in the U.S. FAERS from May 2020 to March 2024 to identify potential adverse event signals. In addition to detecting new signals, compare FAERS findings with clinical trial data to identify discrepancies and validate real-world evidence. The results are expected to support the rational and safe use of Ripretinib in clinical practice and refine its safety profile.

## 2 Materials and methods

### 2.1 Study design and data sources

The FDA Adverse Event Reporting System is a widely accessible database for postmarketing safety monitoring, collecting AEs reports from health professionals, drug manufacturers, and patients. The FAERS database comprises seven data sets: DEMO (patient demographics and management), DRUG (medication details), REAC (adverse event codes), OUTC (outcomes), RPSR (report sources), THER (therapy dates), and INDI (indications for use), along with a category for deleted cases. Data from the FDA’s website are integrated into MySQL 8.0 for comprehensive analysis. We used keywords ‘Ripretinib,’ ‘QINLOCK,’ and ‘DCC 2618’ to extract data from FAERS for statistical analysis, minimizing errors from incomplete data. This research utilizes data extracted from the FAERS database, spanning from May 2020 to March 2024.

The Medical Dictionary for Regulatory Activities (MedDRA) version 24.0 was used to encode AEs in the FAERS database. MedDRA’s terminology is structured into five levels: System Organ Class (SOC), High-Level Group Term (HLGT), High-Level Term (HLT), Preferred Term (PT), and Lowest Level Term (LLT) (Brown et al., 1999). Each Ripretinib adverse event report was sourced from FAERS database records, with SOC and PT levels accurately coded according to MedDRA in our study. Drugs in the FAERS database are categorized into four groups: PS (primary suspect), SS (secondary suspect), C (concomitant), and I (interaction). Key patient outcomes include death (DE), life-threatening events (LT), hospitalization (initial or prolonged) (HO), disability (DS), congenital anomalies (CA), and other significant medical events (OT). We collected data on clinical characteristics including sex, weight, age, reporting region, reporter, reporting duration, and outcomes of AEs associated with Ripretinib.

### 2.2 Statistical analysis

As a commonly used approach in pharmacovigilance study, disproportionality analysis was performed to detect spontaneous signals (Zink et al., 2013). In this study, multiple disproportionality analysis methods, including frequency method and Bayesian method, were used to comprehensively mine the signals of drug-related AEs. Specifically, the frequency method mainly consists of reporting ratio of ratio (ROR) and proportional reporting ratio (PRR), in which ROR can effectively reduce the bias of low-frequency reported events, while PRR performs well in signal screening due to its higher specificity. The Bayesian approach, on the other hand, consists of Bayesian Confidence Propagation Neural Network (BCPNN) and Multi-Item Gamma Poisson Shrinker (MGPS) (Norén et al., 2013). The BCPNN is still capable of effective signal detection when the amount of data is low or missing, and its results are more stable as the number of reports increases, while the MGPS is particularly good at identifying rare adverse reaction signals. The present study uses a combination of these four methods, to expand the scope of signal detection and validation, and to reduce false positives through cross-validation, further improving the detection of rare adverse reactions. An analytical approach was applied to describe the characteristics of each AE report associated with Ripretinib. Disproportionality analysis, a common practice in pharmacovigilance studies, was used to identify potential associations between Ripretinib and all reported AEs. Four key indicators were used to evaluate the potential link between Ripretinib and AEs: the ROR, PRR, BCPNN (Bate et al., 1998; Evans et al., 2001), and MGPS, as outlined in Table 1. Each of the four algorithms was used to identify at least one positive indicator of drug-related AEs, with criteria including a 95% confidence interval (CI) greater than 1, N ≥ 3; PRR ≥ 2, χ^2^ ≥ 4; IC025 > 0, or EBGM05 > 2 (Li et al., 2023; Liu, 2024).

**TABLE 1:** Four major algorithms used for signal detection.

Algorithms	Equation	Criteria
ROR	ROR = ad/b/c	lower limit of 95% CI > 1, N ≥ 3
	95%CI = e^ln(ROR)±1.96(1/a+1/b+1/c+1/d) ^ 0.5^	
PRR	PRR = a (c + d)/c/(a+b)	PRR ≥ 2, χ^2^ ≥ 4, N ≥ 3
	χ^2^ = [(ad-bc) ^ 2](a+b + c + d)/[(a+b) (c + d) (a+c) (b + d)]	
BCPNN	IC = log_2_a (a+b + c + d) (a+c) (a+b)	IC025 > 0
	95%CI = E (IC) ± 2V(IC) ^ 0.5	
MGPS	EBGM = a (a+b + c + d)/(a+c)/(a+b)	EBGM05 > 2
	95%CI = e^ln(EBGM)±1.96(1/a+1/b+1/c+1/d) ^ 0.5^	

Notes: Equation: a, number of reports containing both the target drug and target adverse drug reaction; b, number of reports containing other adverse drug reaction of the target drug; c, number of reports containing the target adverse drug reaction of other drugs; d, number of reports containing other drugs and other adverse drug reactions.

Abbreviations: ROR, reporting odds ratio; 95% CI, 95% confidence interval; PRR, proportional reporting ratio; N, the number of reports; χ2, chi-squared; BCPNN, bayesian confidence propagation neural network; IC, information component; IC025, the lower limit of 95% CI, of the IC; E (IC), the IC, expectations; V(IC), the variance of IC; EBGM, empirical Bayesian geometric mean; EBGM05, the lower limit of 95% CI, of EBGM.

## 3 Results

### 3.1 General characteristics

**FIGURE 1:**
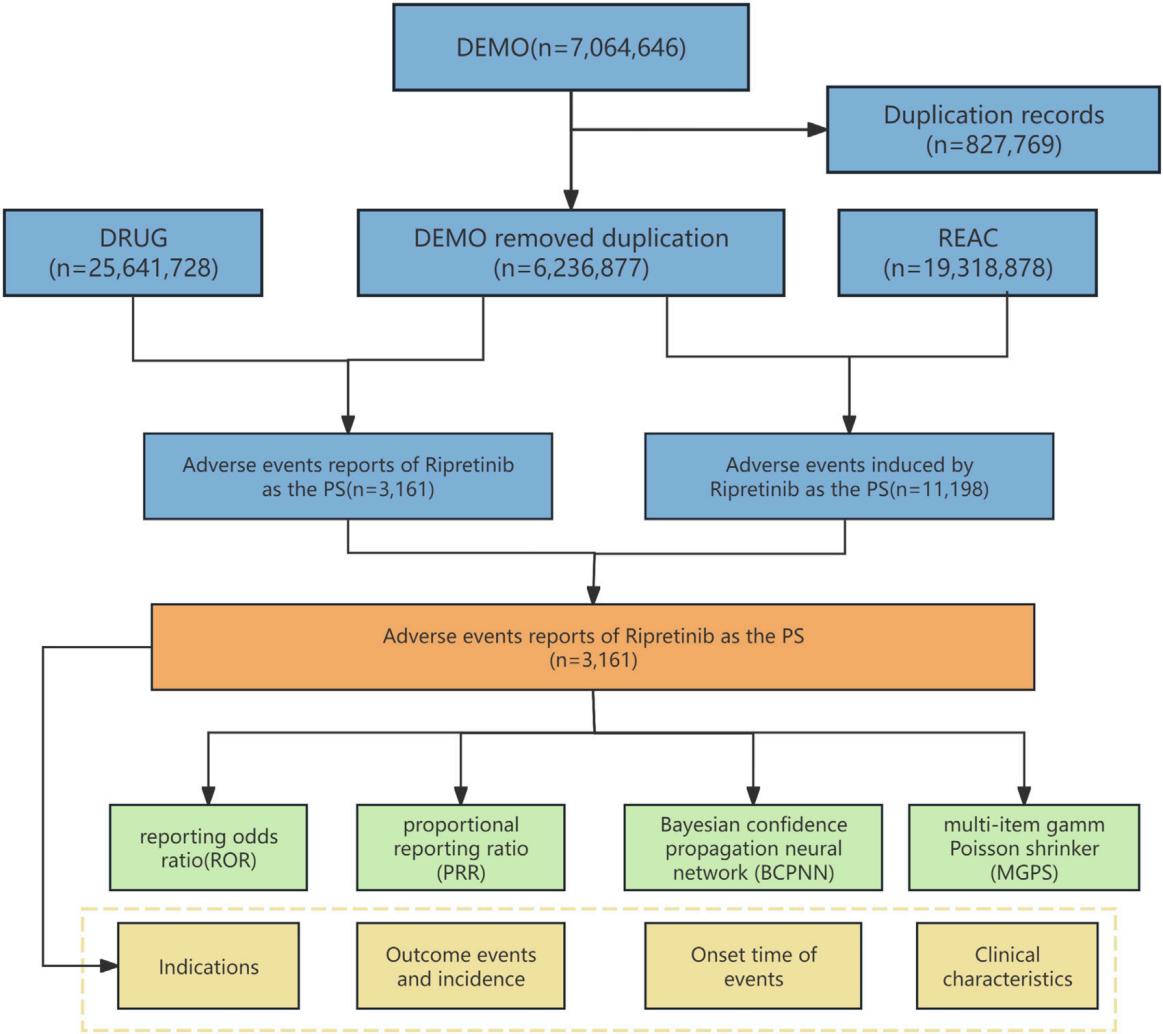
Flowchart of identifying adverse event cases of Ripretinib from the FAERS database.

Between May 2020 and March 2024, the FAERS database received 7,064,646 AE reports, of which 3,161 were related to Ripretinib. Figure 1 provides a flowchart that outlines the process of data extraction and analysis. Table 2 details the characteristics of the Ripretinib-related AE reports submitted. From 2020 to 2024, the annual number of AE reports varied, with the highest in 2023 (34.8%), followed by 2022 (26.3%). Across all reports, males accounted for 53.9% and females 44%.Among the patients, 6.1% weighed between 50 and 100 kg, compared to 0.5% over 100 kg and 0.9% under 50 kg. Additionally, weight information was missing for 92.6% of reports. Patients aged 65–85 years constituted the highest proportion (21.4%), followed by those aged 18–64.9 years (14.9%) and over 85 years (1.2%). The majority of reports originated from the United States (92.9%), followed by France (1.7%), Canada (1.5%), Germany (0.5%), and the UK (0.5%), with the primary reporters being consumers (59.7%) and health professionals (45.7%).

**TABLE 2:** Clinical characteristics of reports with ripretinib from the FAERS database.

Characteristics		Case number (n)	Case proportion (%)
	Number of events	3,161	
Gender	Male	1704	53.9
	Female	1,392	44.0
	Missing	65	2.1
Weight (kg)	<50	27	0.9
	50–100	193	6.1
	>100	15	0.5
	Missing	2,926	92.6
Age (years)	18–64.9	470	14.9
	65–85	678	21.4
	>85	38	1.2
	Missing	1974	62.4
Reporter’s Type of Occupation	Consumer	1887	59.7
	Health profession	1,301	40.1
	Missing	5	0.2
Reported Countries	United States	2,935	92.9
	France	53	1.7
	Canada	46	1.5
	Germany	15	0.5
	Great Britain	15	0.5
Outcome	Death	326	9.6
	Disability	3	0.1
	Hospitalization	597	17.6
	Life-threatening	16	0.5
	Other	497	14.7
	Missing	1945	57.5

### 3.2 Signal detection

**FIGURE 2:**
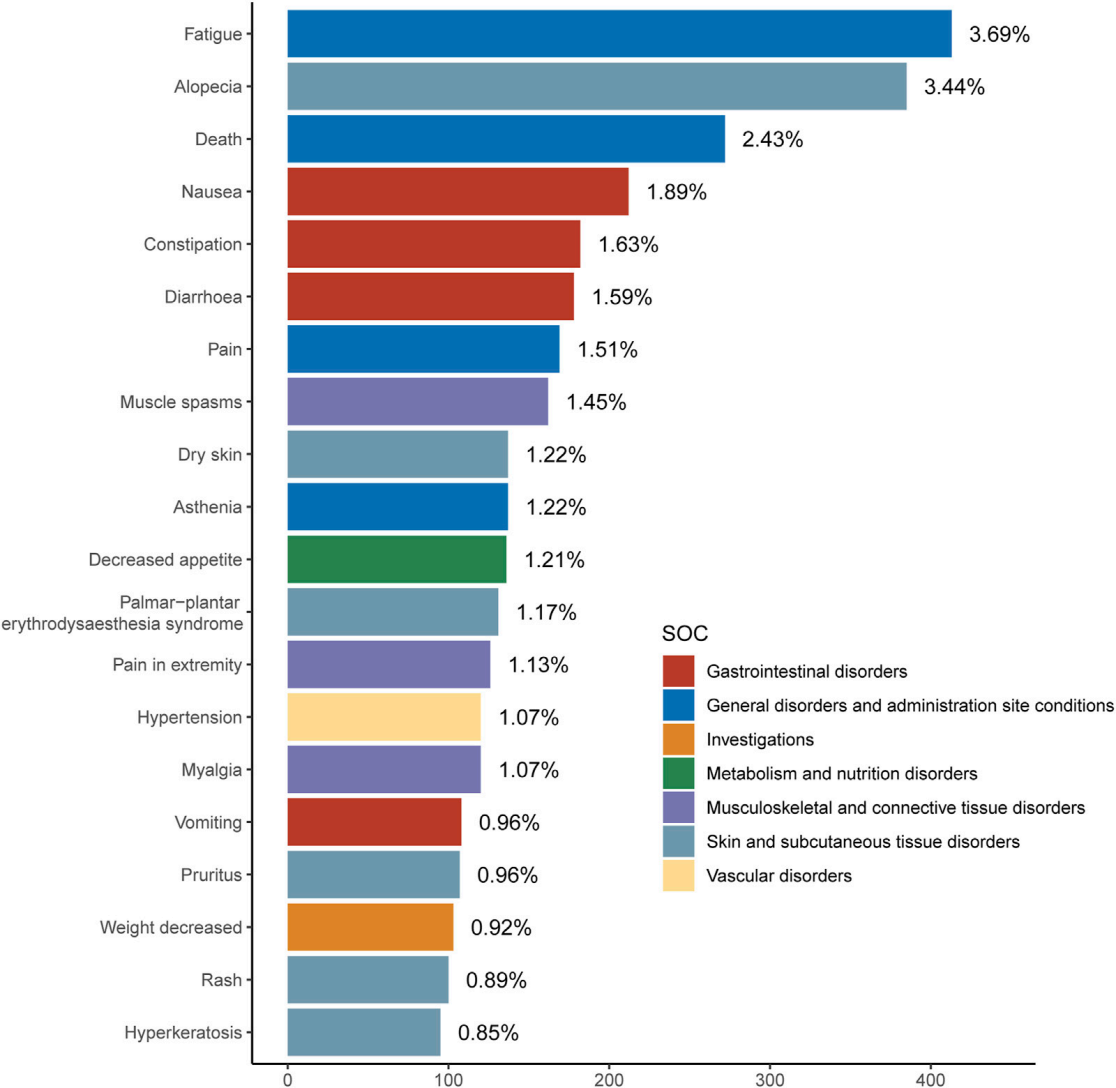
Signal Strength of top20 AEs of Ripretinib at the System Organ Class (SOC) Level in FAERS Database.

**FIGURE 3:**
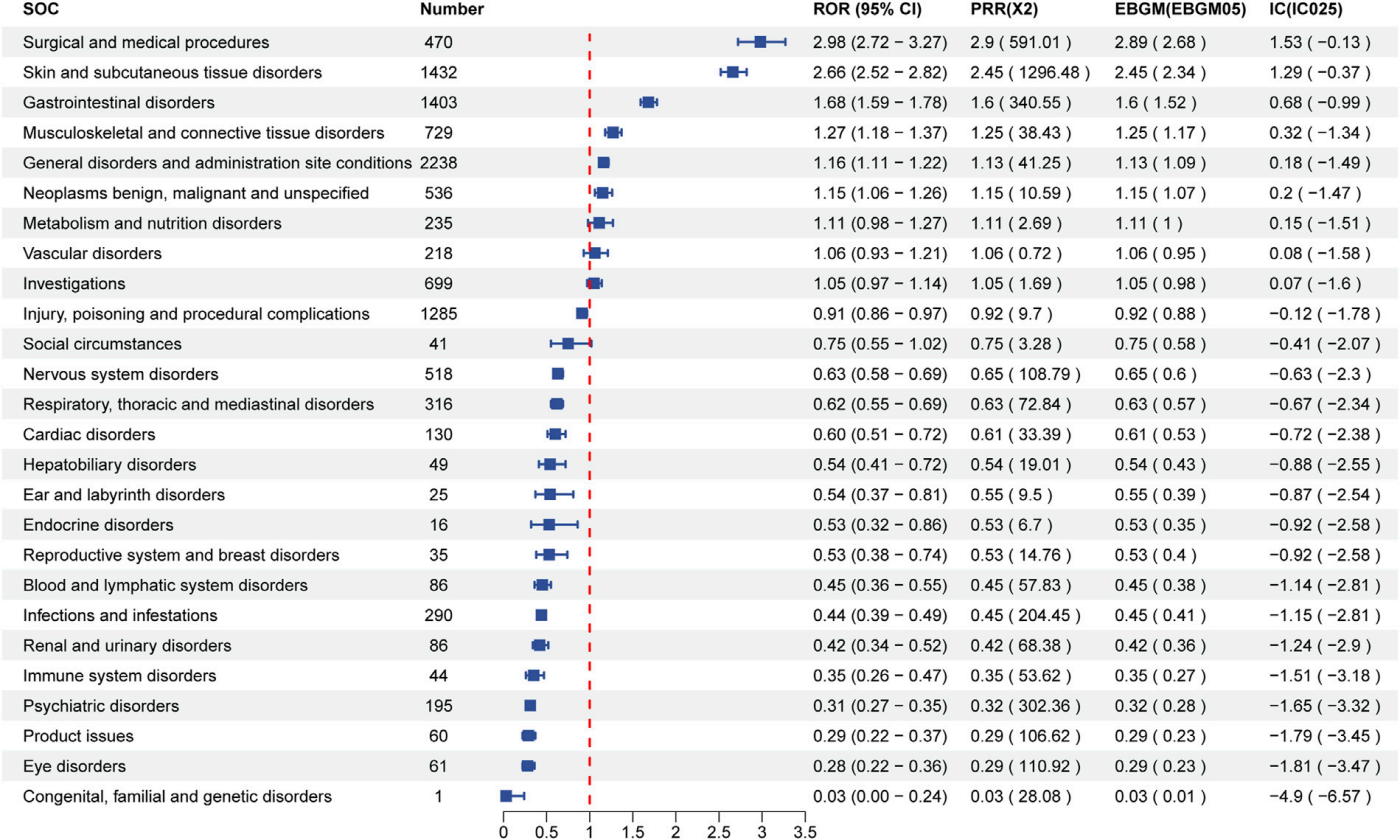
Signal strength of reports of ripretinib at the preferred terms level in FAERS database.

Table 3 and Figure 2 detail the top 20 preferred terms (PTs). The proportions of AEs were as follows: fatigue (3.68%), alopecia (3.43%), diarrhea (3.43%), death (2.42%), nausea (1.89%), constipation (1.62%), pain (1.58%), muscle cramps (1.44%), dry skin (1.22%), decreased appetite (1.21%), palmoplantar erythrodysesthesia (1.16%), limb pain (1.12%), hypertension (1.07%), muscle pain (1.07%), vomiting (0.96%), itching (0.95%), weight loss (0.91%), rash (0.89%), and keratoderma (0.84%). Most positive signals aligned with known AEs listed in the Repitinib drug insert. High-frequency signals, such as fatigue, alopecia, nausea, pain, constipation, myalgia, diarrhea, appetite loss, palmar-plantar erythema syndrome, and vomiting, were all clearly documented in the insert. These findings further confirm the study’s accuracy and practical relevance. Supplementary Table 1 lists the top 40 AEs signal strengths for Ripretinib at the preferred term level, ranked by EBGM. Notably, unexpected significant AEs, including “hepatic neoplasm”, “hair texture abnormal”, “metastases to liver” and “red blood cell count decreased”, were also identified in the label. The results may provide a reference for further updating the AE in the specification of Ripretinib. These findings are particularly concerning as they suggest potential long-term complications of Ripretinib treatment that warrant further investigation and close clinical monitoring. Table 4 and Figure 3 present the signal intensity and reporting of Ripretinib at significant SOC levels (PT > 100). Statistically, Ripretinib induced AEs in 26 SOCs, with the most affected being “surgical and medical procedures”, “skin and subcutaneous tissue disorders”, “gastrointestinal disorders”, “musculoskeletal and connective tissue disorders”, and “neoplasms benign, malignant and unspecified (incl cysts and polyps)”.

**TABLE 3:** Signal strength of top 20 AEs of ripretinib at the preferred terms level in FAERS database.

System organ class (SOC)	Preferred terms	Case Reports(n)	ROR (95% two-sided CI)	PRR (95% two-sided CI)	χ2	IC(IC025)	EBGM (EBGM05)
Gastrointestinal disorders	Nausea	212	1.72 (1.5–1.97)	1.7 (1.57–1.84)	62.2	0.77 (−0.9)	1.7 (1.52)
	Constipation	182	4.76 (4.11–5.52)	4.7 (4.56–4.85)	530.82	2.23 (0.56)	4.69 (4.15)
	Diarrhoea	178	1.52 (1.31–1.76)	1.51 (1.36–1.65)	30.83	0.59 (−1.07)	1.51 (1.33)
	Vomiting	108	1.51 (1.25–1.83)	1.51 (1.32–1.7)	18.67	0.59 (−1.07)	1.51 (1.29)
General disorders and administration site conditions	Fatigue	413	2.9 (2.63–3.2)	2.83 (2.73–2.92)	493.23	1.5 (−0.17)	2.82 (2.6)
	Death	272	1.79 (1.59–2.02)	1.77 (1.65–1.89)	92.23	0.82 (−0.84)	1.77 (1.6)
	Pain	169	1.21 (1.04–1.41)	1.21 (1.06–1.36)	6.3	0.28 (−1.39)	1.21 (1.07)
	Asthenia	137	2.27 (1.92–2.69)	2.25 (2.09–2.42)	95.97	1.17 (−0.49)	2.25 (1.96)
Investigations	Weight decreased	103	2.03 (1.67–2.47)	2.02 (1.83–2.21)	53.35	1.01 (−0.65)	2.02 (1.72)
Metabolism and nutrition disorders	Decreased appetite	136	3.29 (2.78–3.9)	3.26 (3.09–3.43)	213.66	1.7 (0.04)	3.26 (2.83)
Musculoskeletal and connective tissue disorders	Muscle spasms	162	6.03 (5.16–7.05)	5.96 (5.81–6.11)	667.93	2.57 (0.9)	5.94 (5.22)
	Pain in extremity	126	2.61 (2.19–3.11)	2.59 (2.42–2.76)	123.26	1.37 (−0.29)	2.59 (2.23)
	Myalgia	120	5.04 (4.21–6.03)	5 (4.82–5.17)	383.16	2.32 (0.65)	4.98 (4.29)
Skin and subcutaneous tissue disorders	Alopecia	385	11.87 (10.72–13.14)	11.49 (11.39–11.59)	3,675.06	3.51 (1.85)	11.42 (10.49)
	Dry skin	137	5.51 (4.65–6.52)	5.45 (5.29–5.62)	497.86	2.44 (0.78)	5.44 (4.72)
	Palmar-plantar erythrodysaesthesia syndrome	131	31.28 (26.29–37.21)	3 0.92 (30.75–31.1)	3,727.92	4.93 (3.26)	30.4 (26.28)
	Pruritus	107	1.61 (1.33–1.94)	1.6 (1.41–1.79)	24.31	0.68 (−0.99)	1.6 (1.37)
	Rash	100	1.3 (1.07–1.59)	1.3 (1.1–1.49)	6.93	0.38 (−1.29)	1.3 (1.1)
	Hyperkeratosis	95	112.14 (91.06–138.12)	111.2 (9,747.2–111.41)	110.99	6.71 (5.04)	104.52 (87.81)
Vascular disorders	Hypertension	120	3.31 (2.76–3.96)	3.29 (3.11–3.46)	191.06	1.71 (0.05)	3.28 (2.82)

Abbreviations: ROR, reporting odds ratio; CI, confidence interval; PRR, proportional reporting ratio; χ2, chi-squared; IC, information component; EBGM, empirical Bayesian geometric mean; IC025, the lower limit of 95% CI, of the IC; EBGM05, the lower limit of 95% CI, of EBGM.

**TABLE 4:** Signal strength of reports of ripretinib at the system organ class (SOC) level in FAERS database.

System organ class (SOC)	Cases reporting SOC	ROR (95% two-sided CI)	PRR (χ2)	EBGM(EBGM05)	IC(IC025)
Surgical and medical procedures	470	2.98 (2.72–3.27)	2.9 (591.01)	2.89 (2.68)	1.53 (−0.13)
Skin and subcutaneous tissue disorders	1,432	2.66 (2.52–2.82)	2.45 (1,296.48)	2.45 (2.34)	1.29 (−0.37)
Gastrointestinal disorders	1,403	1.68 (1.59–1.78)	1.6 (340.55)	1.6 (1.52)	0.68 (−0.99)
Musculoskeletal and connective tissue disorders	729	1.27 (1.18–1.37)	1.25 (38.43)	1.25 (1.17)	0.32 (−1.34)
General disorders and administration site conditions	2,238	1.16 (1.11–1.22)	1.13 (41.25)	1.13 (1.09)	0.18 (−1.49)
Neoplasms benign, malignant and unspecified	536	1.15 (1.06–1.26)	1.15 (10.59)	1.15 (1.07)	0.2 (−1.47)
Metabolism and nutrition disorders	235	1.11 (0.98–1.27)	1.11 (2.69)	1.11 (1)	0.15 (−1.51)
Vascular disorders	218	1.06 (0.93–1.21)	1.06 (0.72)	1.06 (0.95)	0.08 (−1.58)
Investigations	699	1.05 (0.97–1.14)	1.05 (1.69)	1.05 (0.98)	0.07 (−1.6)
Injury, poisoning and procedural complications	1,285	0.91 (0.86–0.97)	0.92 (9.7)	0.92 (0.88)	−0.12 (−1.78)
Social circumstances	41	0.75 (0.55–1.02)	0.75 (3.28)	0.75 (0.58)	−0.41 (−2.07)
Nervous system disorders	518	0.63 (0.58–0.69)	0.65 (108.79)	0.65 (0.6)	−0.63 (−2.3)
Respiratory, thoracic and mediastinal disorders	316	0.62 (0.55–0.69)	0.63 (72.84)	0.63 (0.57)	−0.67 (−2.34)
Cardiac disorders	130	0.6 (0.51–0.72)	0.61 (33.39)	0.61 (0.53)	−0.72 (−2.38)
Hepatobiliary disorders	49	0.54 (0.41–0.72)	0.54 (19.01)	0.54 (0.43)	−0.88 (−2.55)
Ear and labyrinth disorders	25	0.54 (0.37–0.81)	0.55 (9.5)	0.55 (0.39)	−0.87 (−2.54)
Endocrine disorders	16	0.53 (0.32–0.86)	0.53 (6.7)	0.53 (0.35)	−0.92 (−2.58)
Reproductive system and breast disorders	35	0.53 (0.38–0.74)	0.53 (14.76)	0.53 (0.4)	−0.92 (−2.58)
Blood and lymphatic system disorders	86	0.45 (0.36–0.55)	0.45 (57.83)	0.45 (0.38)	−1.14 (−2.81)
Infections and infestations	290	0.44 (0.39–0.49)	0.45 (204.45)	0.45 (0.41)	−1.15 (−2.81)
Renal and urinary disorders	86	0.42 (0.34–0.52)	0.42 (68.38)	0.42 (0.36)	−1.24 (−2.9)
Immune system disorders	44	0.35 (0.26–0.47)	0.35 (53.62)	0.35 (0.27)	−1.51 (−3.18)
Psychiatric disorders	195	0.31 (0.27–0.35)	0.32 (302.36)	0.32 (0.28)	−1.65 (−3.32)
Product issues	60	0.29 (0.22–0.37)	0.29 (106.62)	0.29 (0.23)	−1.79 (−3.45)
Eye disorders	61	0.28 (0.22–0.36)	0.29 (110.92)	0.29 (0.23)	−1.81 (−3.47)
Congenital, familial and genetic disorders	1	0.03 (0–0.24)	0.03 (28.08)	0.03 (0.01)	−4.9 (−6.57)

Abbreviations: ROR, reporting odds ratio; CI, confidence interval; PRR, proportional reporting ratio; χ2, chi-squared; IC, information component; EBGM, empirical Bayesian geometric mean.

### 3.3 Time to onset of ripretinib-associated adverse events

**FIGURE 4:**
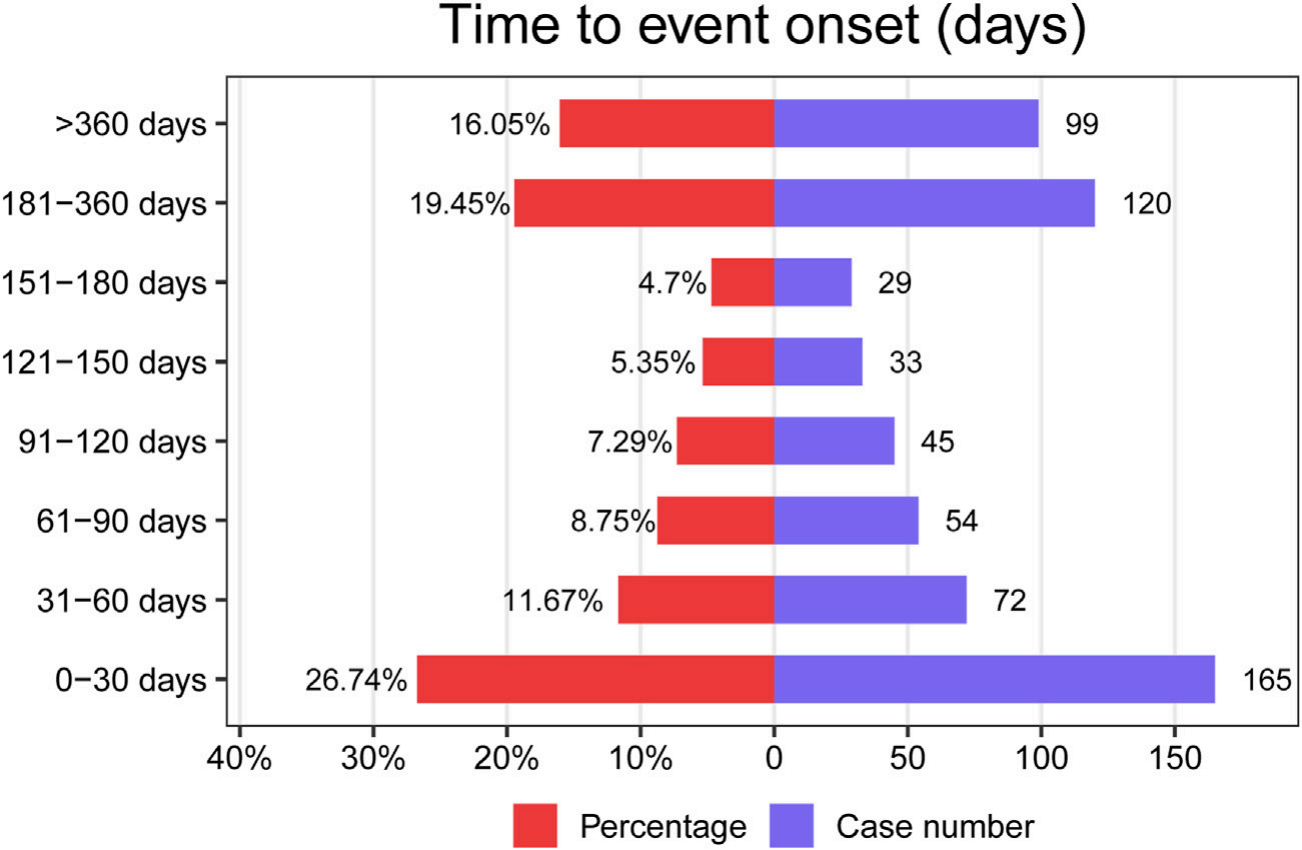
Time to event onset times.

**FIGURE 5:**
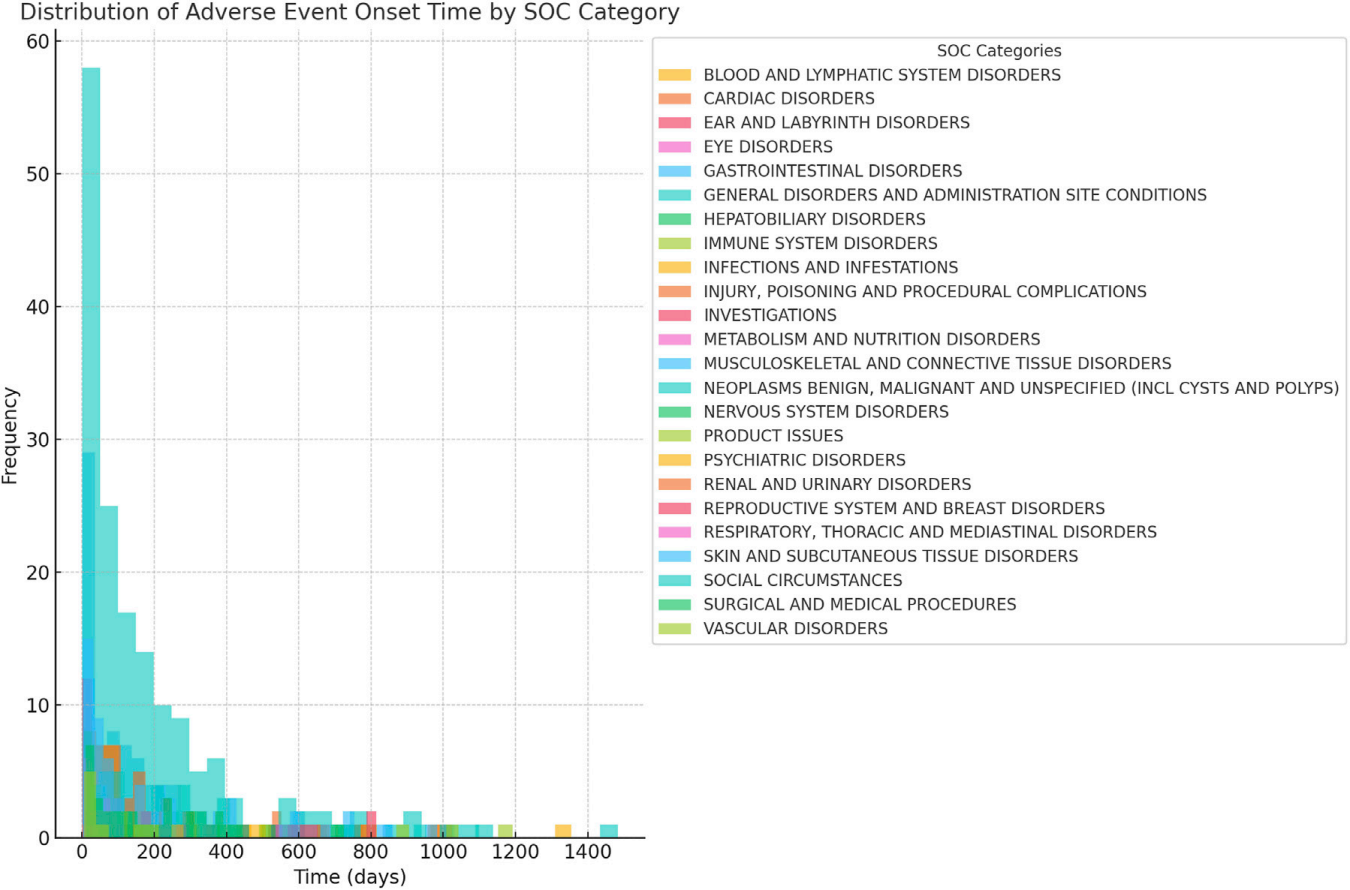
Distribution of time to adverse event at system organ class (SOC).

The onset time of Ripretinib-related AEs was extracted from the database. After excluding reports with inaccurate, missing, or unknown periods, 617 cases of ripretinib-related AEs with reported onset times were analyzed. The median onset time was 99 days, with an interquartile range (IQR) of 27–245 days Figure 4 shows that it was observed that the majority of AEs (n = 165, 26.74%) occurred within the first month after dosing. Thereafter, there was a decreasing trend in AEs over time, over the next 6 months. However, between 6 months and 1 year of dosing, AEs appeared to rise again, and did not decrease significantly after 1 year of drug use. This finding contributes to a better understanding and management of the safety issues associated with Ripretinib, allowing for timely adjustments to the treatment regimen to mitigate adverse effects and improve treatment outcomes. Figure 5 shows the distribution of adverse event onset times for different System Organ Classes. There were significant differences in the time to onset of adverse reactions in different system organ categories. Overall, gastrointestinal disorders occurred over a shorter period of time, usually in the early stages of treatment, whereas ear and eye disorders tended to occur over the long course of treatment.

## 4 Discussion

This represents the first extensive pharmacovigilance analysis of post-marketing AEs related to Ripretinib, based on the FAERS database, offering unprecedented accuracy and detail in describing these AEs.

In our study, a total of 3,161 potential signals were identified. The incidence of AE in males (53.9%) was slightly higher than that in females (44.0%), indicating the gender susceptibility of AE. However, in the global epidemiological statistics of GIST, the gender distribution of GIST patients is quite equal (Søreide et al., 2016). In a cohort study on the effect of age and gender on the tumor-related prognosis of gastrointestinal stromal tumor, it was found that only young women showed better disease-specific survival (Kramer et al., 2015), which may be because the incidence of heterogeneous type 2 GISTs in women is as high as 80%, which has a better prognosis than kinase mutated GIST in adults (so-called type 1 GIST) (Italiano et al., 2012; Miettinen and Lasota, 2014). In addition, some studies have suggested that male gender is a poor prognostic factor for GIST (Fujimoto et al., 2003; Singer et al., 2002), which means that male patients with GIST usually show more severe symptoms or complications. Due to the lack of weight information in 92.6% of the reports, this study did not explore the impact of weight on AE incidence. Individuals aged 65–85 comprised 21.4% of the cases (678 cases) and were more prone to AEs, consistent with mesothelioma studies indicating a median diagnosis age over 60 (Søreide et al., 2016). Significantly, 59.7% of adverse reaction reports were submitted by patients, not healthcare professionals, suggesting that patients may be more proactive in reporting AEs post-Ripretinib use or that there is underreporting by medical staff. As 92.9% of the reports originated from the United States, this may indicate regional or cultural reporting biases, necessitating further investigation.

At the SOC level, general disorders and administration site conditions were the most common AEs, the significant SOCs were surgical and medical procedures and skin and subcutaneous tissue disorders. As shown in Table 3, common AEs included fatigue, alopecia, diarrhea, death, nausea, constipation, pain, muscle cramps and dry skin, which were mostly consistent with the insert and clinical trials frequently cited on Ripretinib’s label and were confirmed significantly in the present study (Blay et al., 2020; Bauer et al., 2022; Lim et al., 2024). Alopecia frequently led to treatment interruptions or dose reductions in patients treated with Ripretinib. According to the INVICTUS study (Blay et al., 2020), the incidence of Alopecia is as high as 49%. And in a bridging study in China of INTRIGUE study (Li et al., 2024), 27% among Asian patients, and most adverse reactions were grade 1 or 2 (Blay et al., 2020). The epidermal growth factor receptor (EGFR) pathway plays a crucial role in hair follicle biology and epidermal homeostasis (Philpott and Kealey, 1994). EGFR is located in the outer root sheath of hair follicles (Nanney et al., 1984) and is essential for the transition from the growth phase to the maturation phase. Inhibition of EGFR can lead to follicle disintegration accompanied by inflammation, which may explain hair loss (Hansen et al., 1997). The study also highlighted significant AE signals in ‘skin and subcutaneous tissue disorders,’ notably keratoderma and dry skin. Rare but severe, there were instances of fatal reactions like cutaneous squamous cell carcinoma and melanoma. Therefore, we found that Ripretinib is associated with a range of dermatologic AEs, probably because of malignant cells and normal skin mucous membrane tissue Shared between signaling pathways, some target molecules (i.e., EGFR] and vascular EGFR [VEGFR]) are also present in the skin (Deutsch et al., 2020; Reyes-Habito and Roh, 2014; American Cancer Society web site, 2024). It is advised to perform dermatological assessments at the start and throughout treatment. Suspicious skin lesions should be excised and evaluated dermatopathologically. Hypertension is commonly reported as an early complication of TKI therapy (Motzer et al., 2023). The underlying mechanism is thought to involve activation of the endothelin-1 pathway and disruption of endothelial cell survival signaling, leading to decreased capillary density and reduced nitric oxide secretion (Kappers et al., 2010; Rini et al., 2011). Therefore, monitoring blood pressure during treatment is recommended, along with appropriate management of hypertension as required. These findings highlight the importance of regular dermatological and cardiovascular monitoring during Ripretinib treatment. Clinicians should consider routine assessments of skin conditions and blood pressure management to mitigate these side effects and improve patient outcomes.

Other AEs of SOCs involved in adverse reactions mentioned in the instructions, including Vascular disorders and Metabolism and nutrition disorders, have corresponding signals detected and verified the reliability of the data in this study. Some other unexpected and new significant AEs signals that were not mentioned in the instruction or regulatory trials, such as ‘hepatic neoplasm’, ‘hair texture abnormal’, ‘metastases to liver’ and ‘red blood cell count decreased’, were detected in our analysis, and the exact induction mechanisms of these AEs remained unclear. These findings emphasise the need for ongoing monitoring and reporting of AEs, particularly those that were not anticipated in the initial clinical trials.

The study found that the majority of AEs (n = 165, 26.74%) occurred within the first month of Riptinib use. However, AEs were again elevated after 6 months of dosing and did not decrease significantly after 1 year. Differences in the time of occurrence of adverse effects in different classes of drugs reflect the metabolic processes of the drug in the body and its mechanism of action on different physiological systems in the long or short term. Haematological, cardiac and ear adverse reactions usually require more stringent monitoring in the middle or later stages of treatment to prevent chronic toxicity or cumulative effects. Gastrointestinal adverse reactions, on the other hand, tend to be short-term side effects that need to be managed promptly at the beginning of treatment. Therefore, future clinical studies should extend the follow-up period to more fully assess the adverse effects of Ripretinib. The results of the study emphasise the importance of vigilance throughout the course of treatment, not just in the initial phase. Furthermore, in our investigation, we only initially investigated the correlation between Ripretinib and AEs, and therefore we set the drug action as the ‘preferred suspect’, i.e., the drug was the single factor associated with AEs. Therefore, our study did not further investigate the effects of multifactorial confounding, including secondary suspects, concomitant medication and multidrug interactions (Zhao et al., 2024).

Despite these measures, significant data inconsistencies or omissions regarding patients’ age, weight, and other basic information persisted. Such gaps can introduce analytical biases, hindering the accurate identification of the optimal target population for therapy. While the use of a large real-world data set and comprehensive data mining techniques offers benefits, it is important to acknowledge several inherent limitations. First, the FAERS database, as a spontaneous reporting system drawing from various countries and professionals, may contain inconsistencies in data quality and completeness, impacting the analysis. Additionally, this analysis did not account for various factors such as potential drug interactions, comorbidities, and concurrent medications that could affect AEs. Another limitation is the inability to confirm exact causality; disproportionality analysis can estimate signal strength but not measure risk or verify causality, underscoring the need for prospective clinical studies. Despite these limitations, our findings provide valuable guidance for medical experts, facilitating detailed follow-ups and monitoring of Ripretinib-related adverse effects.

## 5 Conclusion

Our pharmacovigilance analysis of the FAERS database provides a comprehensive assessment of the safety signals associated with Ripretinib therapy. Attention should be given to new and unexpected AEs during treatment, as some may be life-threatening and require early detection and intervention. Our findings highlight both expected and unexpected AEs associated with Ripretinib treatment, suggesting the need for continued pharmacovigilance. Long-term clinical studies are essential to confirm these signals and refine the drug’s safety profile.

## Data Availability

The original contributions presented in the study are included in the article/Supplementary Material, further inquiries can be directed to the corresponding authors.
