# Supplementary material for: A real-world disproportionality analysis of ripretinib data mining of the public version of FDA adverse event reporting system
Source: Front Pharmacol. 2025 Mar 18;16:1469597. doi: 10.3389/fphar.2025.1469597 (PMC11959022; doi:10.3389/fphar.2025.1469597)
Supplement: Supplementary file 1 [file Table1.docx]

Supplementary Material

# Supplementary Figures and Tables

## Supplementary Table 1

| High Level Term (HLT) | Case Reports | ROR_95 | PRR_95 | IC(IC025) | EBGM(EBGM05) |
| --- | --- | --- | --- | --- | --- |
| HYPERKERATOSIS | 95 | 112.14 ( 91.06 - 138.12 ) | 111.2 ( 9747.2 ) | 6.71 ( 5.04 ) | 104.52 ( 87.81 ) |
| TUMOUR EXCISION | 14 | 100.29 ( 58.49 - 171.94 ) | 100.16 ( 1298.96 ) | 6.57 ( 4.89 ) | 94.72 ( 60.33 ) |
| PALMAR-PLANTAR ERYTHRODYSAESTHESIA SYNDROME | 131 | 31.28 ( 26.29 - 37.21 ) | 30.92 ( 3727.92 ) | 4.93 ( 3.26 ) | 30.4 ( 26.28 ) |
| SKIN HYPERTROPHY | 14 | 29.99 ( 17.67 - 50.88 ) | 29.95 ( 385.08 ) | 4.88 ( 3.21 ) | 29.45 ( 18.93 ) |
| HEPATIC NEOPLASM | 14 | 29.4 ( 17.33 - 49.88 ) | 29.37 ( 377.19 ) | 4.85 ( 3.18 ) | 28.89 ( 18.56 ) |
| CANCER PAIN | 15 | 26.53 ( 15.93 - 44.2 ) | 26.5 ( 362.5 ) | 4.71 ( 3.04 ) | 26.11 ( 17.04 ) |
| MELANOCYTIC NAEVUS | 22 | 25.34 ( 16.62 - 38.61 ) | 25.29 ( 505.84 ) | 4.64 ( 2.97 ) | 24.94 ( 17.53 ) |
| HAIR TEXTURE ABNORMAL | 34 | 17.6 ( 12.55 - 24.68 ) | 17.55 ( 525.27 ) | 4.12 ( 2.45 ) | 17.38 ( 13.09 ) |
| GINGIVAL BLEEDING | 27 | 14.51 ( 9.93 - 21.21 ) | 14.48 ( 336.06 ) | 3.84 ( 2.18 ) | 14.37 ( 10.46 ) |
| ALOPECIA | 385 | 11.87 ( 10.72 - 13.14 ) | 11.49 ( 3675.06 ) | 3.51 ( 1.85 ) | 11.42 ( 10.49 ) |
| METASTASES TO LIVER | 31 | 10.05 ( 7.06 - 14.31 ) | 10.03 ( 250.52 ) | 3.32 ( 1.65 ) | 9.97 ( 7.42 ) |
| BLOOD IRON DECREASED | 25 | 9.56 ( 6.45 - 14.18 ) | 9.54 ( 190.24 ) | 3.25 ( 1.58 ) | 9.5 ( 6.83 ) |
| BLOOD BILIRUBIN INCREASED | 25 | 7.32 ( 4.94 - 10.84 ) | 7.3 ( 135.48 ) | 2.86 ( 1.2 ) | 7.28 ( 5.24 ) |
| NEOPLASM | 16 | 7.08 ( 4.33 - 11.57 ) | 7.07 ( 83.03 ) | 2.82 ( 1.15 ) | 7.04 ( 4.67 ) |
| MUSCLE SPASMS | 162 | 6.03 ( 5.16 - 7.05 ) | 5.96 ( 667.93 ) | 2.57 ( 0.9 ) | 5.94 ( 5.22 ) |
| BLISTER | 53 | 5.58 ( 4.26 - 7.32 ) | 5.56 ( 197.86 ) | 2.47 ( 0.81 ) | 5.55 ( 4.42 ) |
| DRY SKIN | 137 | 5.51 ( 4.65 - 6.52 ) | 5.45 ( 497.86 ) | 2.44 ( 0.78 ) | 5.44 ( 4.72 ) |
| SKIN FISSURES | 19 | 5.02 ( 3.2 - 7.88 ) | 5.01 ( 60.87 ) | 2.32 ( 0.66 ) | 5 ( 3.43 ) |
| MYALGIA | 120 | 5.04 ( 4.21 - 6.03 ) | 5 ( 383.16 ) | 2.32 ( 0.65 ) | 4.98 ( 4.29 ) |
| CONSTIPATION | 182 | 4.76 ( 4.11 - 5.52 ) | 4.7 ( 530.82 ) | 2.23 ( 0.56 ) | 4.69 ( 4.15 ) |
| PAIN OF SKIN | 23 | 4.61 ( 3.06 - 6.94 ) | 4.6 ( 64.68 ) | 2.2 ( 0.53 ) | 4.59 ( 3.26 ) |
| SKIN DISORDER | 27 | 4.06 ( 2.78 - 5.92 ) | 4.05 ( 61.94 ) | 2.02 ( 0.35 ) | 4.04 ( 2.95 ) |
| BLOOD PRESSURE ABNORMAL | 16 | 3.91 ( 2.39 - 6.39 ) | 3.91 ( 34.55 ) | 1.96 ( 0.3 ) | 3.9 ( 2.59 ) |
| BLOOD POTASSIUM DECREASED | 18 | 3.79 ( 2.39 - 6.03 ) | 3.79 ( 36.88 ) | 1.92 ( 0.25 ) | 3.78 ( 2.57 ) |
| HYPERSOMNIA | 17 | 3.53 ( 2.19 - 5.68 ) | 3.52 ( 30.66 ) | 1.81 ( 0.15 ) | 3.52 ( 2.36 ) |
| SKIN EXFOLIATION | 52 | 3.49 ( 2.66 - 4.59 ) | 3.48 ( 91.89 ) | 1.8 ( 0.13 ) | 3.48 ( 2.77 ) |
| RED BLOOD CELL COUNT DECREASED | 19 | 3.37 ( 2.15 - 5.29 ) | 3.36 ( 31.53 ) | 1.75 ( 0.08 ) | 3.36 ( 2.3 ) |
| HYPERTENSION | 120 | 3.31 ( 2.76 - 3.96 ) | 3.29 ( 191.06 ) | 1.71 ( 0.05 ) | 3.28 ( 2.82 ) |
| DECREASED APPETITE | 136 | 3.29 ( 2.78 - 3.9 ) | 3.26 ( 213.66 ) | 1.7 ( 0.04 ) | 3.26 ( 2.83 ) |
| FATIGUE | 413 | 2.9 ( 2.63 - 3.2 ) | 2.83 ( 493.23 ) | 1.5 ( -0.17 ) | 2.82 ( 2.6 ) |
| PAIN IN EXTREMITY | 126 | 2.61 ( 2.19 - 3.11 ) | 2.59 ( 123.26 ) | 1.37 ( -0.29 ) | 2.59 ( 2.23 ) |
| ASTHENIA | 137 | 2.27 ( 1.92 - 2.69 ) | 2.25 ( 95.97 ) | 1.17 ( -0.49 ) | 2.25 ( 1.96 ) |
| WEIGHT DECREASED | 103 | 2.03 ( 1.67 - 2.47 ) | 2.02 ( 53.35 ) | 1.01 ( -0.65 ) | 2.02 ( 1.72 ) |
| DEATH | 272 | 1.79 ( 1.59 - 2.02 ) | 1.77 ( 92.23 ) | 0.82 ( -0.84 ) | 1.77 ( 1.6 ) |
| NAUSEA | 212 | 1.72 ( 1.5 - 1.97 ) | 1.7 ( 62.2 ) | 0.77 ( -0.9 ) | 1.7 ( 1.52 ) |
| PRURITUS | 107 | 1.61 ( 1.33 - 1.94 ) | 1.6 ( 24.31 ) | 0.68 ( -0.99 ) | 1.6 ( 1.37 ) |
| DIARRHOEA | 178 | 1.52 ( 1.31 - 1.76 ) | 1.51 ( 30.83 ) | 0.59 ( -1.07 ) | 1.51 ( 1.33 ) |
| VOMITING | 108 | 1.51 ( 1.25 - 1.83 ) | 1.51 ( 18.67 ) | 0.59 ( -1.07 ) | 1.51 ( 1.29 ) |
| RASH | 100 | 1.3 ( 1.07 - 1.59 ) | 1.3 ( 6.93 ) | 0.38 ( -1.29 ) | 1.3 ( 1.1 ) |
| PAIN | 169 | 1.21 ( 1.04 - 1.41 ) | 1.21 ( 6.3 ) | 0.28 ( -1.39 ) | 1.21 ( 1.07 ) |

**Supplementary Table 1.** The top 40 high level terms (HLT) of AEs evaluated using ROR, PRR, BCPNN, and EBGM according to frequency ranking.
